# Microbial diversity of a full‐scale UASB reactor applied to poultry slaughterhouse wastewater treatment: integration of 16S rRNA gene amplicon and shotgun metagenomic sequencing

**DOI:** 10.1002/mbo3.443

**Published:** 2017-02-23

**Authors:** Tiago Palladino Delforno, Gileno Vieira Lacerda Júnior, Melline F. Noronha, Isabel K. Sakamoto, Maria Bernadete A. Varesche, Valéria M. Oliveira

**Affiliations:** ^1^ Microbial Resources DivisionResearch Center for ChemistryBiology and Agriculture (CPQBA)Campinas University ‐ UNICAMPCampinasSão PauloBrazil; ^2^ Laboratory of Biological ProcessesDepartment of Hydraulics and SanitationEngineering School of São Carlos ‐ University of São Paulo (EESC ‐ USP) Campus IISão CarlosSão PauloBrazil

**Keywords:** anaerobic microbial community, antibiotics resistance genes, genetic potential, granular sludge, virome

## Abstract

The 16S rRNA gene amplicon and whole‐genome shotgun metagenomic (WGSM) sequencing approaches were used to investigate wide‐spectrum profiles of microbial composition and metabolic diversity from a full‐scale UASB reactor applied to poultry slaughterhouse wastewater treatment. The data were generated by using MiSeq 2 × 250 bp and HiSeq 2 × 150 bp Illumina sequencing platforms for 16S amplicon and WGSM sequencing, respectively. Each approach revealed a distinct microbial community profile, with *Pseudomonas* and *Psychrobacter* as predominant genus for the WGSM dataset and *Clostridium* and *Methanosaeta* for the 16S rRNA gene amplicon dataset. The virome characterization revealed the presence of two viral families with *Bacteria* and *Archaea* as host, *Myoviridae,* and *Siphoviridae*. A wide functional diversity was found with predominance of genes involved in the metabolism of acetone, butanol, and ethanol synthesis; and one‐carbon metabolism (*e.g.,* methanogenesis). Genes related to the acetotrophic methanogenesis pathways were more abundant than methylotrophic and hydrogenotrophic, corroborating the taxonomic results that showed the prevalence of the acetotrophic genus *Methanosaeta*. Moreover, the dataset indicated a variety of metabolic genes involved in sulfur, nitrogen, iron, and phosphorus cycles, with many genera able to act in all cycles. BLAST analysis against Antibiotic Resistance Genes Database (ARDB) revealed that microbial community contained 43 different types of antibiotic resistance genes, some of them were associated with growth chicken promotion (*e.g.,* bacitracin, tetracycline, and polymyxin).

## 1 INTRODUCTION

The anaerobic digestion (AD) corresponds to a consolidated technology that is successfully applied in different wastes and influents for the conversion of complex organic compounds to CH_4_ and CO_2_, nutrient recovery (phosphorus and sulfur), pollutant removal and/or energy (CH_4_ and H_2_), and fermentative compound (butyrate, butanol) production (Batstone & Virdis, 2014; Battistoni et al., 1997).

The large AD application is only possible due to the occurrence of complex interactions of microorganisms belonging to Bacteria and Archaea domains (Zinder, 1984). Previous studies have reported that microbial composition of anaerobic digestion sludge is highly diverse (Cabezas et al., 2015). However, information on the key players in anaerobic conditions and their interactions is not comprehensive. These results indicate a high genetic potential that has been poorly explored and open promising perspectives for biotechnological applications.

WGSM sequencing has been shown as an excellent approach to assess the genetic potential of a microbial community, unraveling the phylogenetic composition, metabolic capacity, and functional diversity (Streit & Schmitz, 2004) of community members. However, comprehensive information on the phylogenetic composition is highly dependent on the sequence number and in general the shotgun metagenomic sequencing is not deep enough. On the other hand, WGSM sequencing does not involve the biased amplification of 16S rRNA genes, and although more precise, the organism abundances are still dependent on the DNA extraction and sequencing protocols used (Kalyuzhnaya et al., 2008; Morgan, Darling, & Eisen, 2010).

Although the 16S rRNA gene amplicon sequencing has showed several artifacts due to PCR amplification and sequencing errors, this approach offers a deep sequencing for a large microbial community characterization allowing to detect rare species in complex communities (Quince et al., 2009). For this reason, the integration of 16S rRNA gene amplicon and WGSM sequencing approaches represent a robust strategy for the study of microbiomes. It is important to emphasize that the microbiological knowledge (phylogenetic composition, metabolic capacity, and functional diversity) in AD is fundamental to elect microbial indicators of optimal performance, to understand the microbial behavior in response to environmental disturbance and to optimize the process (Carballa, Regueiro, & Lema, 2015).

The term metagenomics was first used by Handelsman et al. (1998) in a soil microbiome study and the first report associating metagenomics with AD occurred only 10 years later in a deep microbial analysis of a German full‐scale biogas plant treating farm waste (Schlüter et al., 2008). Thenceforward, several studies have examined the AD under a taxonomic and functional perspective, allowing the reconstruction of important metabolic pathways and genomes (Campanaro et al., 2016; Wang et al., 2013; Wirth et al., 2012; Wong et al., 2013) with focus on the understanding of biological interactions and optimization of bioprocesses. Despite the fact that the first report on the application of metagenomics in AD has been from real‐scale reactors, most of the studies have focused on the evaluation of laboratory‐scale reactors. Therefore, scarce investigation has been performed in full‐scale reactors treating real wastewater and these reactors should not be neglected, especially those applied to wastewater treating of industrial activities of global impact like poultry production.

Brazil represents one of main poultry exporters worldwide, with more than 4.2 million tons per year (Facta 2016). This intense poultry production results in wastewater with high polluting potential due mainly to the process and the washing of equipment with high chemical oxygen demand (COD; 1.790–4.760 mg L‐1), oil and grease (114–640 mg L‐1), nitrogen (90–196 mg N L‐1), and phosphorus (22–84 mg L‐1) (de Nardi, Fuzi, & Del Nery, 2008; Del Nery et al., 2013). The AD technology, especially the up‐flow anaerobic sludge blanket (UASB) reactor, has been applied as the main alternative for poultry slaughterhouse wastewater treatment. Apart from the engineering aspects related to the optimization of UASB reactors for treatment of this type of wastewater, several studies have investigated the microbiological potential of the microbiome in granular sludge from UASB reactors, and the results are promising. The high microbial diversity observed (Delforno et al., 2015) represents a high genetic diversity and, consequently, a broad metabolic potential for several bioprocesses. Therefore, a wide application of granular sludge for nitrogen (Moura, Damianovic, & Foresti, 2012) and sulfide removal (Camiloti et al., 2014), hydrogen production (Penteado et al., 2013), anionic surfactant degradation (Okada et al., 2014), polychlorinated biphenyl (PCB) degradation (Gomes et al., 2014), and veterinary antimicrobial removal (Oliveira, Santos‐Neto, & Zaiat, 2015) has been observed.

In this context, the objective of this study was to carry out a robust description of the microbiome in a full‐scale UASB reactor applied to poultry slaughterhouse wastewater treatment. For the in‐depth microbiome survey, an integrated approach combining 16S rRNA gene amplicon and WGSM sequencing was used. In addition to the description of the community composition (Bacteria, Archaea, and Virus), the metabolic processes related to biogeochemical cycles (e.g., sulfur, iron, phosphorus, nitrogen) and antibiotic resistance genes were explored (microbial metabolic potential). In this sense, the robust information on the microbiome composition, functioning, and interactions may guide novel biotechnological applications of the granular sludge present in the UASB reactor under study.

## 2 MATERIAL AND METHODS

### 2.1 Wastewater treatment system of the poultry slaughterhouse

The wastewater treatment system of poultry slaughterhouse is located in Tiête, São Paulo State (Brazil) and consists of four parts: (1) rotary screens to remove feathers and bowels; (2) static screens to remove the fine solids; (3) dissolved‐air flotation system (DAF); and (4) full‐scale UASB reactor. The circular UASB reactor had a flow rate of 975 m^3^ d^−1^, an area of 170 m^2^ and operated under mesophilic condition. The hydraulic retention time (HRT) was 1.02 days. The average influent COD of reactorwas 2,000 mg L^−1^ with 77–88% of removal. Average grease and fats were 308 and 469 mg L^−1^ for effluent and influent, respectively. The total solids (TSS) were 2,133 mg L^−1^ in the effluent and 1,593 in the influent, whereas, the concentration of N‐NH_3_ and P‐PO_4_ in the influent reached 48 mg L^−1^ and 38 mg L^−1^, respectively.

### 2.2 Sampling, DNA extraction, Sequencing, and Preprocessing data

Samples examined in this study were collected from UASB reactor in December 2013. Initially, granular sludge was collected in 20 l high‐density polyurethane bottles from UASB reactor. In the laboratory, 25 ml of the granular sludge was transferred to 50 ml Falcon tubes and stored at −20°C until analyzed. The surplus, present in the high‐density polyurethane bottles, was stored at 4°C to be used as an inoculum for other anaerobic bioreactor studies.

For the in‐depth microbiome characterization, two strategies were adopted: (i) the sequencing of 16S rRNA gene amplicons for phylogenetic characterization and (ii) metagenomic sequencing for functional diversity characterization.

The sample for phylogenetic characterization was sequenced using the MiSeq 2 × 250 bp and DNA was extracted using a modified phenol–chloroform protocol described by Griffiths et al. (2000). The 16S rRNA genes were amplified using the primer set S‐D‐Bact‐0341‐b‐S‐17 (5′‐CCTACGGGNGGCWGCAG‐3′) and S‐D‐Bact‐0785‐a‐A‐21 (5′‐GACTACHVGGGTATCTAATCC‐3′), flanking the V3 and V4 hypervariable regions, as described by Klindworth et al. (2013). 16S amplicon sequencing was performed at the Animal Biotechnology Lab, Department of Animal Science (ESALQ/USP Piracicaba, Brazil) on a MiSeq platform following the manufacturer's guidelines. On the other hand, whole metagenome was sequenced using the HiSeq 2 × 150 bp and DNA was extracted using the PowerSoil^®^ DNA Isolation kit (MoBio Laboratories, Inc., Carlsbad, CA, USA). WGSM sequencing was performed at MR DNA (www.mrdnalab.com, Shallowater, TX, USA) on a HiSeq platform following the manufacturer's guidelines.

The differences between the platforms used for each sample include the desired fragment size, quantity of data generated, and cost of sequencing. For the phylogenetic diversity, fragment size is highly important to increase the reliability of the evolutionary relatedness among sequences used as OTUs, whereas for the evaluation of the functional diversity the amount of generated data is important to increase the coverage. In both cases, the DNA quality was assessed by an ND‐2000 spectrophotometer (Nanodrop Inc., Wilmington, DE), using a ratio of 260/280 nm >1.8, and agarose gel electrophoresis.

All datasets in FastQ format were uploaded to MG‐RAST server (http://metagenomics.nmpdr.org/) version 3.5 (Meyer et al., 2008) for preprocessing and annotation. The artificial replicate sequences were removed according to Gomez‐Alvarez, Teal, and Schmidt (2009), whereas the low‐quality sequences were removed using the modified Dynamic Trim (Cox, Peterson, & Biggs, 2010); the default parameters were adopted.

The sequence data are available at MG‐RAST server access number 4633385.3(Amplicon_PS) and 4626733.3 (WGS_whole).

### 2.3 Microbial community structure and composition (phylogenetic characterization)

**Figure 1:**
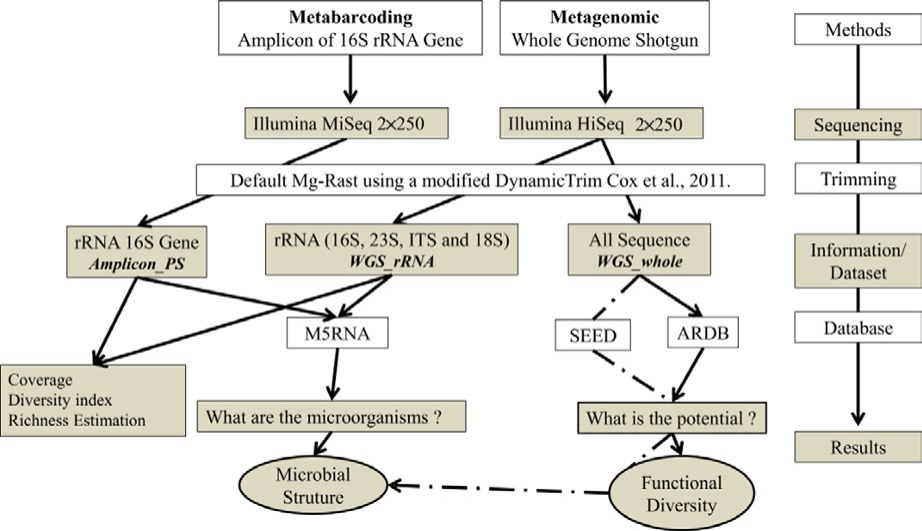
Pipeline of methods and analyses. The sequence data are available at MG‐RAST server under the access number 4633385.3 (Amplicon_PS) and 4626733.3 (WGS_whole)

The microbial community structure and composition of the UASB reactor sludge was evaluated using two approaches: (1) the 16S rRNA gene sequences derived from the amplicon sequencing were used against the M5RNA (Nonredundant multisource ribosomal RNA annotation) database (Meyer et al., 2008); (2) the 16S, 23S, ITS, and 18S rRNA sequences derived from the metagenome sequencing were extracted and analyzed against the M5RNA database (WGS_rRNA*;* Figure 1). The parameters adopted in both analyses were as follows: max. E‐value cutoff = 1e^−5^, min. % identity cutoff = 60% and min. alignment length cutoff = 15. Moreover, taxonomic assignment of metagenomic sequence (WGS_whole) was performed against the SEED database with default parameters (Overbeek et al., 2005). In both cases (using the M5RNA and SEED database), the best hit classification was used to visualize the results.

#### 2.3.1 Diversity analyses

Coverage and alpha diversity indices (dominance, Simpson, Shannon, and Chao‐1) were calculated using the PAST software version 3.07 (Hammer, Harper, & Ryan, 2001). Thus, the .biom files of each sample generated by the MG‐RAST (QIIME report; Caporaso et al., 2010) were the input files for the PAST software.

### 2.4 Functional analysis

Functional classification of WGS_whole‐sequence dataset was conducted by MG‐RAST annotation pipeline using the SEED subsystems database. The data were compared using a maximum e‐value of 1e^−5^, a minimum identity of 60%, and a minimum alignment length of 15 amino acids for proteins.

#### 2.4.1 Metabolic mapping

Some specific metabolic mappings, such as carbon, nitrogen, phosphorus, sulfur, iron, protein, and aromatic compounds, were investigated in details. Sequences assigned to each of the metabolic mappings mentioned above were extracted from SEED dataset and affiliated to the taxonomic groups harboring the most related function (protein) to provide a picture of the microbial community likely involved in such specific metabolic pathways. The taxonomic assignment was performed using the SEED database with default parameters and the data were visualized using the best hit classification for each feature.

Moreover, putative antibiotic resistance genes (ARGs) were searched in the WGS_whole dataset against the Antibiotic Resistance Database (ARDB), which include nonredundant genes, using BLASTX at a cutoff E value ≤10^−5^. A read was identified as an ARG‐like sequence according to its best BLASTx hit with amino acid identity of ≥90% and alignment length of ≥25 amino acids (Kristiansson et al., 2011; Yang et al., 2013).

## 3 RESULTS

### 3.1 Sequencing statistics

In total, 52,826,969 and 293,825 sequences, with average lengths of 233 ± 78 bp and 454 ± 14 bp, were obtained for WGSM sequencing and 16S amplicon sequencing, respectively. After trimming, 66.4% and 34.0% of sequences were removed due to the low quality and the average lengths were reduced to 213 ± 91 bp and 336 ± 156 bp, for WGSM sequencing and 16S amplicon sequencing, respectively. The values of GC‐content were around 53–54% for both samples.

### 3.2 Taxonomic characterization of UASB reactor microbiome

The number of rRNA genes extracted from WGSM dataset was 211,795 sequences (1.0% of total sequences; Table 1). On the other hand, for the 16S amplicon dataset, 191,804 sequences were used for taxonomic assignment.

**Table 1:** Diversity analyses indices and estimations. Amplicon_PS → sequencing of 16S rRNA gene amplicons. WGS_rRNA → rRNA (16S, 23S, ITS, and 18S) sequence extracted from metagenomic sequencing

	Amplicon_PS	WGS_rRNA
Numbers of sequences	191,804	211,795
Diversity index		
Shannon_H	3.67 ± 0.01	3.32 ± 0.01
Richness Estimation		
Chao‐1	1,124 ± 55	1,204 ± 62
rRNA data		
OTU number (observed richness)	880	845
Singletons	203	246
Coverage		
Good′s Coverage	99.93%	99.88%

The total number of OTUs (97% similarity cutoff) and singletons obtained were 845–880 and 246–203, for WGS_rRNA and 16S amplicon datasets, respectively. The highest number of OTUs and the lowest number of singletons were found in the 16S amplicon dataset. The values of Shannon_H and Chao‐1 ranged from 3.32 to 3.67 and 1,204 to 1,124, for WGS_rRNA and 16S amplicon datasets, respectively. Slight differences were observed in the microbial structure of the UASB reactor when comparing the WGS_whole_SEED and WGS_rRNA_M5RNA. For the domain level, the relative abundance obtained were 89.6–96.6% for Bacteria and 1.0–0.2% for Archaea, using the SEED and M5RNA databases. On the other hand, the microbial composition from 16S amplicon dataset showed 84.0% for Bacteria and 6.5% for Archaea domains (data not shown).

**Figure 2:**
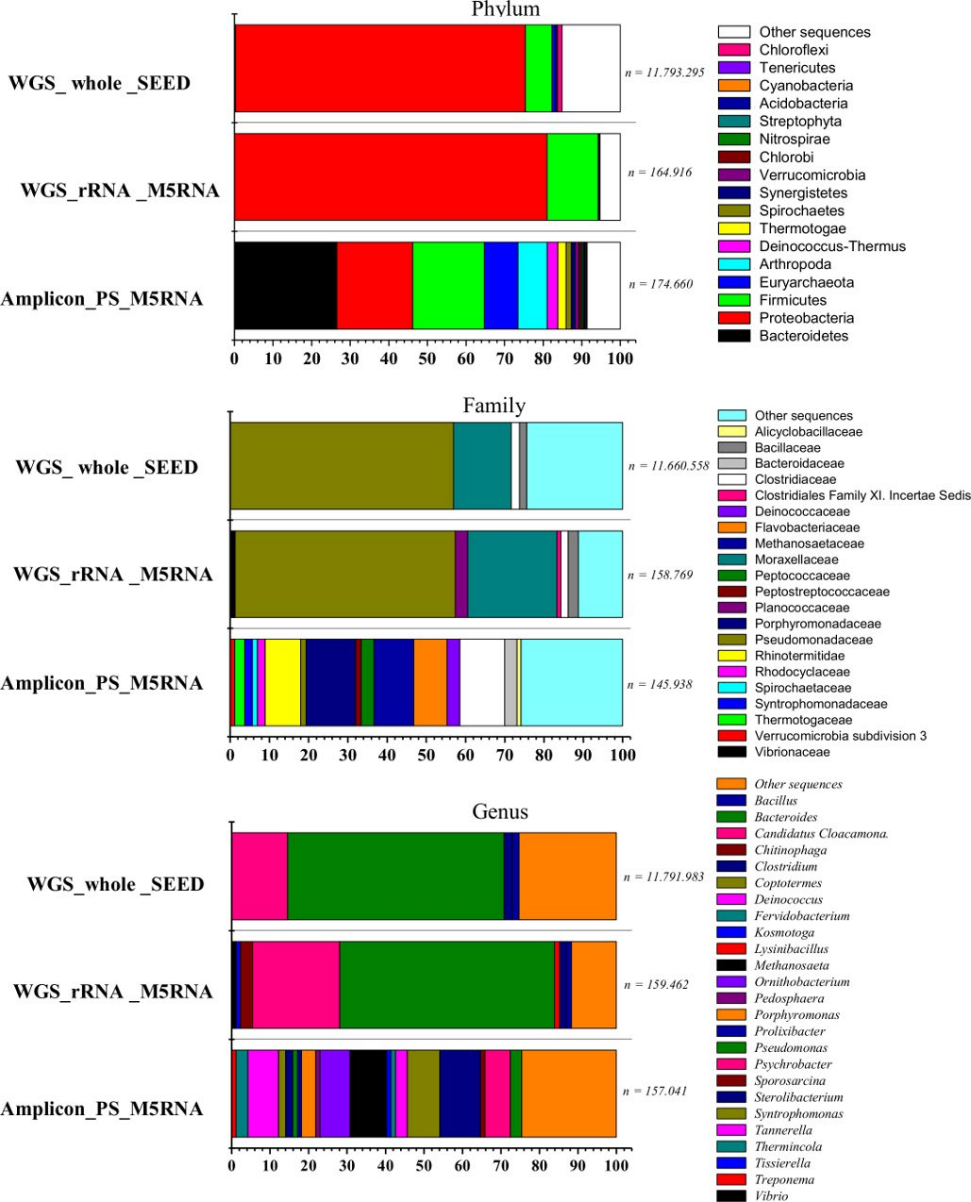
Taxonomic affiliation of reads. *WGS_rRNA* _M5RNA = rRNA gene sequences against the M5RNA database. *Amplicon_PS*_M5RNA = 16S rRNA gene amplicons against the M5RNA database. *WGS_whole* _SEED = all sequences against the SEED database

At the phylum level, 36, 29, and 37 distinct phyla were observed for Amplicon_PS_M5RNA, WGS_rRNA_M5RNA and WGS_whole_SEED, respectively. Protebacteria and Firmicutes were the most representative phyla in WGS_rRNA___M5RNA and WGS_whole_SEED datasets (Figure 2); whereas for the Amplicon_PS_M5RNA dataset, Bacteroidetes was the most abundant phylum, followed by Proteobacteria and Firmicutes. The Euryarchaeota phylum was the only representative (0.25–8.7% relative abundance) for the Archaea domain in all datasets.

At the genus level, a high abundance of *Pseudomonas* (55.8–56.2%)*, Psychrobacter* (14.5–22.6%)*, Sporosarcina* (3.2%)*, Clostridium* (1.8–10.6%), and *Bacillus* (1.3–1.8%) were observed in WGS_rRNA_M5RNA and WGS*_*whole*_*SEED datasets (Figure 2). On the other hand, many different genera with similar abundances were observed in the *Amplicon_*PS_M5RNA dataset, as follows: *Clostridium* (10.5%), *Methanosaeta* (9.5%), *Tannerella* (8.0%), *Ornithobacterium* (7.8%), *Candidatus Cloacomonas* (6.6%), *Porphyromonas* (3.7%), *Deinococcus* (3.0%), and *Thermincola* (3.0%).

**Figure 3:**
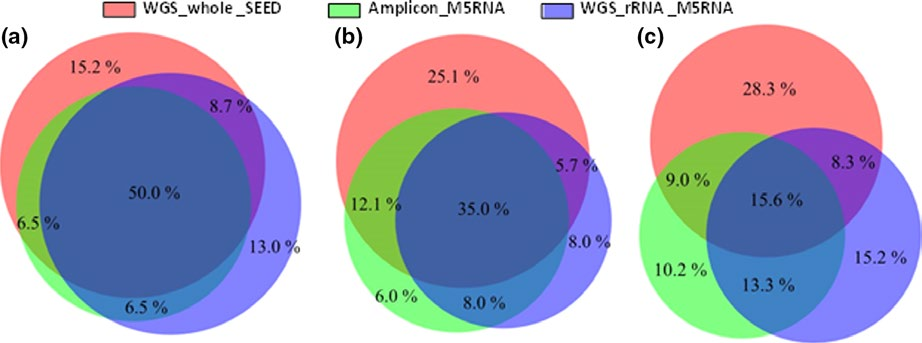
Proportional Venn diagram at Phylum (a), Family (b), and Genera (c) levels

The proportional Venn diagram showed taxonomic overlap percentages from 24% to genus level comparing WGS_whole_SEED and WGS_rRNA _M5RNA datasets. The comparison between datasets containing only rRNA sequences (WGS_rRNA_M5RNA and Amplicon_PS_M5RNA) revealed taxonomic overlap percentages ranging from 29% to 57%, with the lowest overlap value observed at the genus level (Figure 3).

The virome was analyzed from the metagenomic datasets (WGS_whole) using the SEED database (Table 2). Only 0.13% of the total sequences were related to viral genotypes, and the most abundant taxonomies were Microviridae (67.4%), Myoviridae (20.7%), Siphoviridae (9.2%), and Podoviridae (0.5%). Among these, the families Microviridae and Podoviridae have the Bacteria domain as the unique host accounting over 68% of relative abundance; whereas only 30% may use microorganism belong the Archaea domain as host.

**Table 2:** Virus families from WGS_whole_SEED dataset annotated by SEED database through MG‐RAST server

Viral Family	Number of Sequence	Relative Abundance	host
Baculoviridae	159	1.0%	Eukarya
Microviridae	11,224	67.4%	Bacteria
Mimiviridae	58	0.3%	Eukarya
Myoviridae	3,447	20.7%	Bacteria, Archaea
Phycodnaviridae	19	0.1%	Eukarya
Siphoviridae	1,535	9.2%	Bacteria, Archaea
Podoviridae	88	0.5%	Bacteria
Others	112	0.7%	–

### 3.3 Functional profile of UASB reactor microbiome

**Figure 4:**
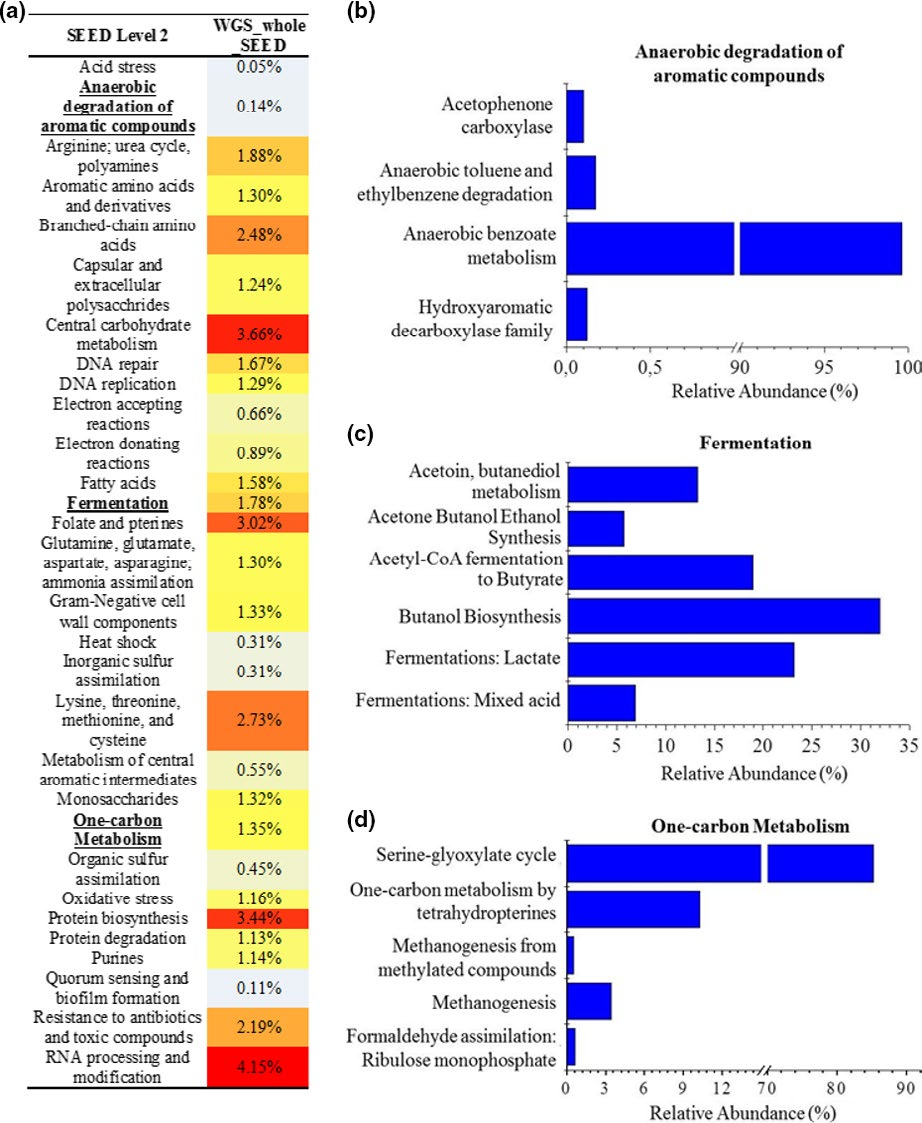
WGS_whole dataset annotated by SEED subsystems database through MG‐RAST server. (a) Heat map of relative abundances of major level 2 SEED (gray for underestimated and red for overestimated abundance). (b – d) Bar plots of level 3 SEED derived from level 2 of anaerobic degradation of aromatic compounds (b), fermentation (c), and one‐carbon metabolism (d) with normalized data

The main Level 1 subsystems were clustering‐based subsystems (13.5%), carbohydrates (12.3%) and amino acids and derivatives (11.3%; Table S1). Inside the carbohydrates category, the subsystems related to central carbohydrate metabolism (3.6%), such as dehydrogenase complexes, glycolysis and gluconeogenesis and pyruvate metabolism II: acetogenesis from pyruvate were the most represented in the dataset. Another important function inside the carbohydrates category was fermentation (1.8%), for example: acetyl‐CoA fermentation to butyrate, acetone butanol ethanol synthesis, and butanol biosynthesis (Figure 4a–c and Table S2).

In the category of one‐carbon metabolism (Level 2 SEED), the relative abundance reached 1.3% (Figure 4A; Table S2). The most abundant metabolic type in this category were serine‐glyoxylate cycle (85.2%), followed by one‐carbon metabolism by tetrahydropterines (10.2%), methanogenesis (3.3%), and methanogenesis from methylated compounds (0.5%; Figure 4d and Table S2). Moreover, functional enzyme‐encoding genes for the methanogenesis pathways were identified and annotated based on the database extracted from KEGG modules. Based on the results, the most prevalent methanogenesis pathway was acetoclastic (43.7%) followed by methylotrophic (34.7%) and hydrogenotrophic (21.6%; Table S3).

In the metabolism of aromatic compounds, 0.14% of reads were related to the anaerobic degradation of aromatic compounds with emphasis on anaerobic benzoate metabolism (99.0%; Figure 4a–b), specifically the function Acetyl‐CoA acetyltransferase.

The cell wall and capsule (4.4%) and quorum sensing and biofilm formation (0.12%) categories, that are likely involved in the formation and maintenance of flocs and biofilms (e.g., granular sludge present in UASB reactors), were found. The main functions in these categories were gram‐negative cell wall components (1.4%), capsular and extracellular polysaccharides (1.2%) and biofilm adhesion biosynthesis (0.06%).

Proteins assigned to iron, nitrogen, sulfur, phosphorus, and potassium metabolisms were found with relative abundance ranging from 0.5% to 2.3% (Tables S1 and S2). In the sulfur metabolism, sequences were assigned to organic and inorganic sulfur assimilation, alkanesulfonate assimilation, sulfur oxidation, and sulfate reduction‐associated complexes; whereas in the nitrogen metabolism, sequences were mainly related to ammonia assimilation, nitrate and nitrite ammonification, and denitrification.

**Figure 5:**
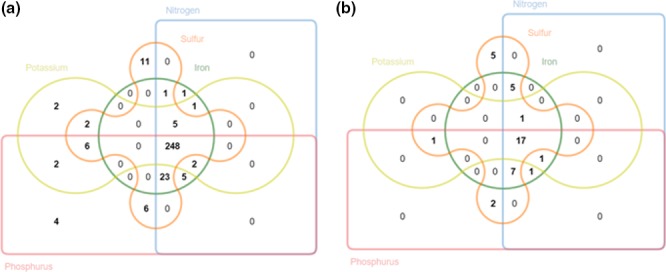
Venn diagram showing the taxonomic affiliation at genus level of whole‐metagenome‐derived reads involved in sulfur, nitrogen, iron, phosphorus, and potassium cycle. (a) Bacteria and (b) Archaea domain

Taxonomic inferences of the genes involved in biogeochemical cycles (sulfur, nitrogen, iron, phosphorus, and potassium) indicated that 248 different bacterial genera showed enzymatic machinery for all biogeochemical cycles (Figure 5a and b). For the domain Archaea, most of the genera (17) are involved in all biogeochemical cycles (Figure 5b). However, only five genera (*Metallosphaera*;* Thermofilum*;* Thermoproteus*;* Ferroplasma;* and *Picrophilus*) showed enzymatic machinery for the sulfur cycle.

### 3.4 Antibiotic Resistance Genes and their host organisms in the UASB reactor microbiome

**Figure 6:**
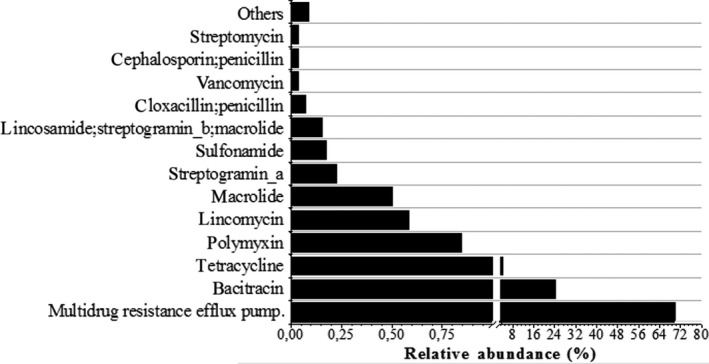
Distribution of reads among the classes of antibiotic resistance genes (ARGs) in the WGS_whole dataset annotated by Antibiotic Resistance Database (ARDB)

In total, 43 different ARGs were found in the UASB reactor microbiome, representing 0.03% of total metagenomics sequences (Figure 6 and Table S4). The top five dominant ARGs types corresponded to multidrug, bacitracin, tetracycline, lincomycin, and polymyxin genes, accounting for over 95% of the total ARGs sequences. The last four ARGs cited are related to nontherapeutic antibiotics, and it can be used as chicken growth promoters. On the other hand, vancomycin resistance (*van*RG) genes, an important therapeutic antibiotic, were found accounting for 0.03% of all ARGs determined. The taxonomic assignment of ARG sequences showed high predominance of the phylum Proteobacteria (91.7%), followed by Firmicutes (3.5%) and Bacteroidetes (0.6%); whereas the main genera were *Pseudomonas* (75.7%), *Burkholderia* (7.1%), *Psychrobacter* (3.9%), and *Moraxella* (3.4%; Table 3 and Table S4).

**Table 3:** Taxonomic classification of sequences related to antibiotics resistance genes (ARGs) from WGS_whole dataset using the SEED database through MG‐RAST server

Taxonomy	Relative Abundance
Bacteroidetes	0.61%
* Bacteroides*	0.37%
* Prevotella*	0.24%
Firmicutes	3.49%
* Bacillus*	0.10%
* Clostridium*	0.48%
* Enterococcus*	0.91%
* Lactobacillus*	0.23%
* Staphylococcus*	0.46%
* Streptococcus*	1.31%
Proteobacteria	91.75%
* Acinetobacter*	0.57%
* Burkholderia*	7.12%
* Campylobacter*	0.13%
* Escherichia*	0.17%
* Klebsiella*	0.10%
* Methylobacillus*	0.63%
* Moraxella*	3.44%
* Pseudomonas*	75.66%
* Psychrobacter*	3.93%
Others	4.16%

## 4 DISCUSSION

Each approach (16S rRNA gene amplicon and WGSM sequencing) revealed a distinct microbial community profile. Probably, these differences observed are a result of the combination of biases introduced during PCR amplification of 16S rRNA gene, different DNA extraction procedures and different high‐throughput sequencing platforms. Differences in microbial composition comparing 16S rRNA amplicon and WGSM sequencing approaches were also obtained by Shah et al. (2010). The authors carried out a systematic comparison using 33 metagenomic datasets of human‐associated bacterial communities and 71 datasets derived from 16S rRNA amplicon sequencing and observed significant differences between the community structures due to the distinct experimental approaches used, suggesting that sample replicates and dataset sizes had smaller influence in the microbial profile than the DNA extraction methods or sequencing protocols. Additionally, De bautista‐ los Santos et al. (2016) using high throughput sequencing of 16S rRNA amplicons for microbial characterization of drink water observed that bias and variability inherent to the PCR amplification and sequencing steps is significant enough to mask differences between bacterial communities from replicate samples. In addition, the average size of the reads obtained in this study (454 bp for 16S amplicon sequencing and 233 bp for WGSM sequencing) and the hypervariable regions analyzed differed between both strategies, potentially leading to different accuracy levels in the phylogenetic affiliation and distinct microbial community profiles (Wang et al., 2007). Poretsky et al. (2014), studying the planktonic microbial community, observed that at phylum level the taxonomic composition in the 16S rRNA library was similar to its corresponding metagenome. On the other hand, at the genus level a large amount of variation between the libraries was observed. This is probably due to the fact that many genera with distinct metabolisms are usually encompassed by the same phylum, and thus important information about variation in particular community members among different communities remains inaccessible. However, it is interesting to emphasize that with the combined use of different tools, more taxonomies could be observed.

The Venn diagram showed the proportion of shared and exclusive taxa found when using the different approaches for phylogenetic inference. Overlap values were similar when comparing datasets derived from the whole‐genome shotgun metagenomic sequencing (WGS_whole_SEED and WGS_rRNA _M5RNA) and datasets containing only rRNA sequences (WGS_rRNA_M5RNA and Amplicon_PS_M5RNA).

The study of viral community in anaerobic wastewater treatment is scarce. However, the viral community may play an important role in methanogenic digestion, since the Archaea (e.g., *Methanosaeta*) may be a favorable target to viral attack (Chien et al., 2013). Therefore, viral infection might explain the decrease in methane production, in upsets and process failures with no obvious explanation (Kroeker et al., 1979). Tamaki et al. (2012) evaluated the viral community in the different stages of wastewater treatment plants, named activated sludge and anaerobic digestion. In the activated sludge the predominant viral families were Podoviridae (45.2%), Siphoviridae (26.5%), and Myoviridae (22.1%). On the other hand, in the anaerobic digestion the order of predominance changed, as follows: Siphoviridae (42.4%), Myoviridae (36.0%), and Podoviridae (16.2%). It is worth to highlight that Siphoviridae and Myoviridae (high presence in the anaerobic digestion where methanogens are abundant) can have Archaea as host, whereas Podoviridae (high presence in the activated sludge) can use only Bacteria as host. In this study, similar results were obtained, with the predominance of Myoviridae (20.7%) and Siphoviridae (9.2%), the exception was the Microviridae family, which was not detected by Tamaki et al. (2012).

The functional profile observed for the UASB reactor microbiome was similar to the one obtained by Lv et al. (2015) when analyzing global functions in the anaerobic‐anoxic reactor applied to denitrifying phosphorus removal. The main functions inside the carbohydrates category and clustering‐based subsystems indicate the microbial genetic potential for fermentative processes. In the category of one‐carbon metabolism, similar values (~1.77% and 2.33%) were observed by Yang et al. (2013), when analyzing the sludge metagenome from full‐scale anaerobic digesters operated in municipal wastewater treatment plants, and by Guo et al. (2015), for the metagenome of anaerobic digester applied to the treatment of activated sludge waste and the production of methane for biofuel.

The abundance of sequences related to the methanogenesis process reached ~3.8% in the UASB reactor microbiome, whereas Guo et al. (2015) observed 9.0%. It is worth mentioning that the main goal of the wastewater treatment system under study is the treatment of organic load from poultry slaughterhouses and not the production of methane for biofuel. The current knowledge recognizes three pathways for methanogenesis named acetoclastic (acetate is reduced to CH_4_), hydrogenotrophic (CO_2_ is reduced to CH_4_), and methylotrophic (methyl group is reduced to CH_4;_ Liu & Whitman, 2008). The prevalence of acetotrophic methanogenic pathways was also consistent with the taxonomic results, which indicated *Methanosaeta* (~9.5% of relative abundance) as the most abundant methanogenic group from the Amplicon_ M5RNA dataset. On the other hand, the hydrogenotrophic (*Methanoculleus*,* Methanolinea,* and *Methanoegula*) and methylotrophic (*Methanosarcina*,* Methanomethylovorans*) methanogenic genera showed 0.24% and 0.15% of relative abundance from the Amplicon_M5RNA dataset, respectively. The prevalence of acetoclastic pathway for methane production in AD has also been observed by Guo et al. (2015) and Yang et al. (2013).

The abundance of reads related to the metabolism of aromatic compounds and resistance to antibiotics and toxic compounds indicates the genetic potential of the microbial community to survive in environments with fluctuating levels of contaminants and stress, in addition to their potential for the application in bioremediation processes. Sanapareddy et al. (2009), evaluating industrial and medical wastewaters, observed 5.0% of aromatic compound metabolism compared to 1.8% observed in this study. These results suggest that the type of wastewater treatment employed may be a key factor in functional gene distribution.

The highest abundance observed was related to iron metabolism, probably due to the selection exerted by the iron present in the poultry blood. Differently, Guo et al. (2015) observed only 0.45% from full‐scale anaerobic reactor treating the waste of activated sludge, suggesting that the presence of iron in poultry blood has probably favored this type of metabolism.

The sequences assigned to the nitrogen and sulfur metabolism suggest the potential of the biomass from UASB reactor treating poultry slaughterhouse to be used as inoculum for different processes, such as: fermentative production, aromatic compound degradation, and sulfur, iron, nitrogen, and phosphorus removal. Moreover, the presence of proteins assigned to the resistance of cobalt‐zinc‐cadmium and arsenic might be biotechnologically explored. Except for the nitrogen metabolism, the relative abundance of reads related to sulfur, phosphorus, and potassium metabolisms were slightly higher than that obtained by Guo et al. (2015) and Yang et al. (2013) when analyzing the metagenome from full‐scale anaerobic digesters.

Taxonomic inference of genes involved in biogeochemical cycles (sulfur, nitrogen, iron, phosphorus, and potassium) suggested that many microbial genera have enzymatic machinery for all biogeochemical cycles. According to Zumstein, Moletta, and Godon (2000) many microbes play the same role in the same biogeochemical cycles, strategy known as functional redundancy that makes the anaerobic digestion process robust and plastic. This strategy is considered as an insurance to maintain ecosystem functions under changing environmental conditions (McMahon, Martin, & Hugenholtz, 2007). The finding of a high number of microbial genera harboring genes involved in all biogeochemical cycles in the UASB reactor microbiome reinforces the importance of functional redundancy within the anaerobic digestion.

Similar conclusion was obtained by Langer et al. (2015) describing the functional redundancy and structural changes of microbial communities involved in laboratory‐scale continuously stirred tank reactors treating maize silage in cofermentation with sugar beet silage. Although the authors observed structural changes according to the different mixtures of maize and sugar beet silage, similar biogas production rates were obtained for equal organic loading rates. Vanwonterghem et al. (2014), operating mesophilic reactors with HRT of 10 days and supplied with a model α‐cellulose, observed similar results for the main steps in AD (hydrolysis, fermentation, and methanogenesis), with multiple phylogenetically diverse populations associated with each step.

Although ARGs have been described in chicken feces (Li et al., 2015), studies of such resistance genes in full anaerobic systems applied to the treatment of poultry slaughterhouse wastewater are scarce. In this study, 0.03% of total metagenomic sequences were related to antibiotic resistance genes. Li et al. (2015) observed higher numbers of ARG sequences in wastewater from livestock farms (0.5%–3.0%). However, ARGs frequency found in this study is similar to the one in drinking water (0.01%–0.05%), soil (0.02%), sediment (0.004–0.03%), and river water (0.02–0.03%; Li et al., 2015).

The frequencies of ARGs are likely associated with the antibiotics used as veterinary medicine, including growth promoters. Previous authors have described that the use of nontherapeutic antibiotics as growth promoters enhanced the abundance of ARGs due to the selective pressure on the microbial community. Looft et al. (2012) observed an increase in ARGs abundance in the swine microbiome after 14 days under antibiotic feeding. Similar results were obtained by Li et al. (2015) when comparing feces from commercially grown chicks with those from 20‐day‐ to 80‐day‐old ones. Thus, the presence of different ARGs types in the poultry slaughterhouse wastewater treatment is probably a consequence of the antibiotics added into the chicken food/water.

The presence of vancomycin ARGs deserves attention since it represents the last line of defense against some bacterial strains resistant to most antibiotics (e.g., *Streptococcus, Enterococcus*). Other authors have found vancomycin resistance genes in swine manure and human feces samples, but in low abundance (0.016%–0.18%). The presence of vancomycin resistance gene it might represent the uncontrolled use of the antibiotic and/or spread of the resistance gene by mobile elements along the microbial community (coselection and co‐occurrence).

In summary, the integration of 16S rRNA gene and metagenome sequencing approaches allowed the in‐depth access of the UASB reactor microbiome, revealing different microbial community profiles and reinforcing the need for integrating multiple tools for microbial community investigation. Analysis of the metagenomic dataset showed the prevalence of *Pseudomonas, Psychrobacter,* and *Sporosarcina* members. On the other hand, the 16S rRNA sequencing approach revealed a higher global diversity and did not show the prevalence of any specific genus. The viral community was mainly composed of the families Microviridae, Myoviridae, and Siphoviridae, with Bacteria and Archaea members as hosts. A wide functional diversity was found in the UASB reactor microbiome, representing a high genetic potential to be further explored in biotechnology such as anaerobic degradation of aromatic compounds, fermentation, protein degradation, and one‐carbon metabolism. Regarding the methanogenic metabolism, genes related to the acetotrophic pathways were more abundant, corroborating the taxonomic results that showed the prevalence of the acetotrophic genus *Methanosaeta*. Moreover, the data indicated the genetic potential of the inoculum for other bioprocesses, such as the removal of sulfur and nitrogen, with many genera able to play a role in all cycles. Different types of antibiotic resistances genes (ARGs) were found, which are likely associated with the antibiotics used as growth chicken promoters and suggesting their uncontrolled use. Combined data gathered in the present work unraveled a robust description of the microbiome (community structure and functional diversity) in a full‐scale UASB reactor and provided fundamental information about the bioprocess in AD that can be further explored in biotechnology.

## CONFLICT OF INTEREST

None declared.
