# Supplementary material for: Microbial diversity of a full‐scale UASB reactor applied to poultry slaughterhouse wastewater treatment: integration of 16S rRNA gene amplicon and shotgun metagenomic sequencing
Source: Microbiologyopen. 2017 Feb 23;6(3):e00443. doi: 10.1002/mbo3.443 (PMC5458456; doi:10.1002/mbo3.443)
Supplement: Supplementary file 1 [file MBO3-6-na-s001.docx]

Table S1: Relative abundance of reads in Level 1 SEED from WGS_whole dataset using the SEED database through MG‑RAST server.

| **Level 1 SEED** | **Relative Abundance** | **Nº of Reads** |
| --- | --- | --- |
| Clustering-based subsystems | 13.465% | 2,413,754 |
| Carbohydrates | 12.268% | 2,199,268 |
| Amino Acids and Derivatives | 11.316% | 2,028,563 |
| Miscellaneous | 7.374% | 1,321,894 |
| Cofactors, Vitamins, Prosthetic Groups, Pigments | 6.124% | 1,097,813 |
| Protein Metabolism | 6.059% | 1,086,106 |
| RNA Metabolism | 4.840% | 867,658 |
| Cell Wall and Capsule | 4.341% | 778,194 |
| Fatty Acids, Lipids, and Isoprenoids | 3.687% | 660,960 |
| DNA Metabolism | 3.666% | 657,120 |
| Membrane Transport | 3.313% | 593,821 |
| Virulence, Disease and Defense | 2.830% | 507,347 |
| Stress Response | 2.496% | 447,404 |
| Respiration | 2.421% | 433,920 |
| Nucleosides and Nucleotides | 2.393% | 428,973 |
| Iron acquisition and metabolism | 2.197% | 393,765 |
| Metabolism of Aromatic Compounds | 1.831% | 328,295 |
| Regulation and Cell signaling | 1.691% | 303,082 |
| Motility and Chemotaxis | 1.292% | 231,627 |
| Phosphorus Metabolism | 1.193% | 213,878 |
| Phages, Prophages, Transposable elements, Plasmids | 1.117% | 200,232 |
| Cell Division and Cell Cycle | 1.017% | 182,379 |
| Nitrogen Metabolism | 0.953% | 170,874 |
| Sulfur Metabolism | 0.943% | 169,074 |
| Potassium metabolism | 0.614% | 109,975 |
| Secondary Metabolism | 0.294% | 52,755 |
| Dormancy and Sporulation | 0.236% | 42,261 |
| Photosynthesis | 0.030% | 5,414 |
|  | | |
