# Supplementary material for: Microbial diversity of a full‐scale UASB reactor applied to poultry slaughterhouse wastewater treatment: integration of 16S rRNA gene amplicon and shotgun metagenomic sequencing
Source: Microbiologyopen. 2017 Feb 23;6(3):e00443. doi: 10.1002/mbo3.443 (PMC5458456; doi:10.1002/mbo3.443)
Supplement: Supplementary file 2 [file MBO3-6-na-s002.docx]

| **SEED subsystems**  Table S2: SEED subsystems (level 2 and 3) from WGS_whole dataset using the SEED database through MG‑RAST server. | **Relative Abundance** | **Nº of Reads** |
| --- | --- | --- |
| **Central carbohydrate metabolism** | **3.6565%** | **655,472** |
| Pyruvate metabolism II: acetyl-CoA, acetogenesis from pyruvate | 0.4854% | 87,017 |
| TCA Cycle | 0.4471% | 80,151 |
| Glycolysis and Gluconeogenesis | 0.4199% | 75,270 |
| Dehydrogenase complexes | 0.3901% | 69,925 |
| Glycolysis and Gluconeogenesis, including Archaeal enzymes | 0.3271% | 58,631 |
| Entner-Doudoroff Pathway | 0.3135% | 56,203 |
| Pyruvate metabolism I: anaplerotic reactions, PEP | 0.2987% | 53,543 |
| Pentose phosphate pathway | 0.2261% | 40,526 |
| Pyruvate Alanine Serine Interconversions | 0.1915% | 34,322 |
| Glyoxylate bypass | 0.1694% | 30,368 |
| Methylglyoxal Metabolism | 0.1353% | 24,260 |
| Glycolate, glyoxylate interconversions | 0.0826% | 14,806 |
| Peripheral Glucose Catabolism Pathways | 0.0725% | 12,995 |
| Pyruvate:ferredoxin oxidoreductase | 0.0554% | 9,935 |
| HPr kinase and hprK operon in Gram-positive organisms | 0.0258% | 4,633 |
| Dihydroxyacetone kinases | 0.0159% | 2,851 |
| Ethylmalonyl-CoA pathway of C2 assimilation | 0.0002% | 36 |
| **Resistance to antibiotics and toxic compounds** | **2.1908%** | **392,724** |
| Cobalt-zinc-cadmium resistance | 0.6515% | 116,789 |
| Copper homeostasis | 0.3047% | 54,628 |
| Multidrug Resistance Efflux Pumps | 0.2630% | 47,155 |
| Resistance to fluoroquinolones | 0.1799% | 32,244 |
| BlaR1 Family Regulatory Sensor-transducer Disambiguation | 0.1686% | 30,219 |
| Multidrug efflux pump in Campylobacter jejuni (CmeABC operon) | 0.1338% | 23,987 |
| Beta-lactamase | 0.0836% | 14,988 |
| Methicillin resistance in Staphylococci | 0.0689% | 12,345 |
| Arsenic resistance | 0.0651% | 11,670 |
| MexE-MexF-OprN Multidrug Efflux System | 0.0575% | 10,310 |
| Copper homeostasis: copper tolerance | 0.0471% | 8,435 |
| Bile hydrolysis | 0.0351% | 6,298 |
| The mdtABCD multidrug resistance cluster | 0.0321% | 5,746 |
| Adaptation to d-cysteine | 0.0302% | 5,418 |
| Erythromycin resistance | 0.0153% | 2,736 |
| Zinc resistance | 0.0141% | 2,534 |
| Cadmium resistance | 0.0101% | 1,814 |
| Resistance to chromium compounds | 0.0095% | 1,704 |
| Fosfomycin resistance | 0.0049% | 884 |
| Lysozyme inhibitors | 0.0042% | 744 |
| Resistance to Vancomycin | 0.0035% | 636 |
| Mercury resistance operon | 0.0031% | 561 |
| Streptothricin resistance | 0.0028% | 499 |
| Mercuric reductase | 0.0013% | 240 |
| Aminoglycoside adenylyltransferases | 0.0006% | 107 |
| Streptococcus pneumoniae Vancomycin Tolerance Locus | 0.0001% | 18 |
| Multidrug Resistance Operon mdtRP of Bacillus | 0.0001% | 10 |
| Polymyxin Synthetase Gene Cluster in Bacillus | 0.0000% | 4 |
| MexA-MexB-OprM Multidrug Efflux System | 0.0000% | 1 |
| **Fermentation** | **1.7836%** | **319,740** |
| Acetyl-CoA fermentation to Butyrate | 0.5701% | 102,200 |
| Acetone Butanol Ethanol Synthesis | 0.4130% | 74,038 |
| Butanol Biosynthesis | 0.3384% | 60,665 |
| Fermentations: Mixed acid | 0.2363% | 42,360 |
| Acetoin, butanediol metabolism | 0.1233% | 22,107 |
| Fermentations: Lactate | 0.1025% | 18,370 |
| **One-carbon Metabolism** | **1.3475%** | **241,562** |
| Serine-glyoxylate cycle | 1.1482% | 205,829 |
| One-carbon metabolism by tetrahydropterines | 0.1382% | 24,767 |
| Methanogenesis | 0.0457% | 8,193 |
| Formaldehyde assimilation: Ribulose monophosphate pathway | 0.0086% | 1,549 |
| Methanogenesis from methylated compounds | 0.0068% | 1,224 |
| **Protein degradation** | **1.1291%** | **202,408** |
| Proteolysis in bacteria, ATP-dependent | 0.5684% | 101,892 |
| Proteasome bacterial | 0.2093% | 37,516 |
| Aminopeptidases | 0.1155% | 20,702 |
| Protein degradation | 0.0756% | 13,559 |
| Metallocarboxypeptidases | 0.0730% | 13,084 |
| Putative TldE-TldD proteolytic complex | 0.0653% | 11,712 |
| Omega peptidases | 0.0090% | 1,610 |
| Dipeptidases | 0.0073% | 1,316 |
| Proteasome archaeal | 0.0031% | 553 |
| Serine endopeptidase | 0.0023% | 413 |
| Metalloendopeptidases | 0.0002% | 42 |
| Proteasome eukaryotic | 0.0001% | 9 |
| **Electron donating reactions** | **0.8889%** | **159,349** |
| Respiratory Complex I | 0.2768% | 49,624 |
| Respiratory dehydrogenases 1 | 0.2043% | 36,631 |
| Hydrogenases | 0.1524% | 27,315 |
| Na(+)-translocating NADH-quinone oxidoreductase and rnf-like group of electron transport complexes | 0.0697% | 12,487 |
| NiFe hydrogenase maturation | 0.0652% | 11,694 |
| Succinate dehydrogenase | 0.0641% | 11,484 |
| H2:CoM-S-S-HTP oxidoreductase | 0.0321% | 5,755 |
| Formate dehydrogenase | 0.0116% | 2,077 |
| CO Dehydrogenase | 0.0055% | 983 |
| Coenzyme F420-H2 dehydrogenase (methanophenazine) | 0.0029% | 517 |
| Energy-conserving hydrogenase (ferredoxin) Ech | 0.0027% | 480 |
| Coenzyme F420 hydrogenase | 0.0017% | 300 |
| Methanophenazine hydrogenase | 0.0000% | 2 |
| **Electron accepting reactions** | **0.6602%** | **118,345** |
| Anaerobic respiratory reductases | 0.2550% | 45,705 |
| Terminal cytochrome C oxidases | 0.1401% | 25,118 |
| Terminal cytochrome oxidases | 0.1123% | 20,134 |
| Terminal cytochrome d ubiquinol oxidases | 0.0728% | 13,044 |
| Terminal cytochrome O ubiquinol oxidase | 0.0344% | 6,165 |
| Ubiquinone Menaquinone-cytochrome c reductase complexes | 0.0270% | 4,835 |
| Tetrathionate respiration | 0.0088% | 1,579 |
| Terminal AA3-600 quinol oxidase | 0.0052% | 925 |
| trimethylamine N-oxide (TMAO) reductase | 0.0045% | 806 |
| fumarate respiration, periplasmic | 0.0002% | 34 |
| **Metabolism of central aromatic intermediates** | **0.5535%** | **99,224** |
| Protocatechuate branch of beta-ketoadipate pathway | 0.1706% | 30,588 |
| Homogentisate pathway of aromatic compound degradation | 0.1306% | 23,414 |
| Catechol branch of beta-ketoadipate pathway | 0.0864% | 15,487 |
| Salicylate and gentisate catabolism | 0.0747% | 13,390 |
| Central meta-cleavage pathway of aromatic compound degradation | 0.0532% | 9,542 |
| N-heterocyclic aromatic compound degradation | 0.0266% | 4,777 |
| 4-Hydroxyphenylacetic acid catabolic pathway | 0.0113% | 2,026 |
| **Organic sulfur assimilation** | **0.4542%** | **81,415** |
| Alkanesulfonate assimilation | 0.2057% | 36,877 |
| Alkanesulfonates Utilization | 0.0660% | 11,825 |
| Utilization of glutathione as a sulphur source | 0.0630% | 11,290 |
| Taurine Utilization | 0.0615% | 11,030 |
| L-Cystine Uptake and Metabolism | 0.0580% | 10,393 |
| **Inorganic sulfur assimilation** | **0.3067%** | **54,984** |
| Inorganic Sulfur Assimilation | 0.3067% | 54,983 |
| F420-dependent sulfite reductase | 0.0000% | 1 |
| **Heat shock** | **0.3063%** | **54,905** |
| Heat shock dnaK gene cluster extended | 0.3063% | 54,905 |
| **Anaerobic degradation of aromatic compounds** | **0.1397%** | **25,040** |
| Anaerobic benzoate metabolism | 0.1391% | 24,941 |
| Anaerobic toluene and ethylbenzene degradation | 0.0002% | 43 |
| Acetophenone carboxylase 1 | 0.0002% | 31 |
| Hydroxyaromatic decarboxylase family | 0.0001% | 25 |
| **Quorum sensing and biofilm formation** | **0.1136%** | **20,367** |
| Biofilm Adhesin Biosynthesis | 0.0562% | 10,080 |
| Quorum Sensing: Autoinducer-2 Synthesis | 0.0338% | 6,056 |
| Quorum sensing regulation in Pseudomonas | 0.0131% | 2,354 |
| Acyl Homoserine Lactone (AHL) Autoinducer Quorum Sensing | 0.0035% | 627 |
| Quorum sensing in Yersinia | 0.0021% | 378 |
| Biofilm formation in Staphylococcus | 0.0020% | 361 |
| Autoinducer 2 (AI-2) transport and processing (lsrACDBFGE operon) | 0.0020% | 360 |
| Quorum-sensing in Vibrio | 0.0008% | 144 |
| Symbiotic colonization and sigma-dependent biofilm formation gene cluster | 0.0000% | 7 |
| **Phosphate metabolism** | **0.0997%** | **17,879** |
| PhoR-PhoB two-component regulatory system | 0.0997% | 17,879 |
| **Acid stress** | **0.0457%** | **8,201** |
| Acid resistance mechanisms | 0.0457% | 8,189 |
| Glutamate transporter involved in acid tolerance in Streptococcus | 0.0001% | 12 |
| **Adhesion** | **0.0343%** | **6,153** |
| Streptococcus pyogenes recombinatorial zone | 0.0249% | 4,456 |
| Adhesion of Campylobacter | 0.0071% | 1,280 |
| Adhesins in Staphylococcus | 0.0020% | 352 |
| Mediator of hyperadherence YidE in Enterobacteria and its conserved region | 0.0004% | 64 |
| Accessory colonization factor | 0.0000% | 1 |
