# Supplementary material for: Microbial diversity of a full‐scale UASB reactor applied to poultry slaughterhouse wastewater treatment: integration of 16S rRNA gene amplicon and shotgun metagenomic sequencing
Source: Microbiologyopen. 2017 Feb 23;6(3):e00443. doi: 10.1002/mbo3.443 (PMC5458456; doi:10.1002/mbo3.443)
Supplement: Supplementary file 3 [file MBO3-6-na-s003.docx]

| **Pathways for Methanogenesis** | **Relative Abundance** | **Relative Abundance** |
| --- | --- | --- |
|  | normalized | without normalize |
| **CO2 => methane** | **21.57%** | **0.11%** |
| **Amino Acids and Derivatives** | **6.56%** | **0.03%** |
| Creatine and Creatinine Degradation | 6.56% | 0.03% |
| **Carbohydrates** | **15.01%** | **0.08%** |
| Methanogenesis | 15.01% | 0.08% |
| **Acetate => methane** | **43.73%** | **0.23%** |
| **Amino Acids and Derivatives** | **29.84%** | **0.16%** |
| Ketoisovalerate oxidoreductase | 14.24% | 0.07% |
| Lysine degradation | 0.04% | 0.0001% |
| Lysine fermentation | 4.19% | 0.02% |
| Threonine anaerobic catabolism gene cluster | 11.37% | 0.06% |
| **Carbohydrates** | **13.89%** | **0.07%** |
| Methanogenesis | 13.89% | 0.07% |
| **Methanol => methane** | **15.17%** | **0.08%** |
| **Amino Acids and Derivatives** | **2.19%** | **0.01%** |
| Creatine and Creatinine Degradation | 2.19% | 0.01% |
| **Carbohydrates** | **12.98%** | **0.07%** |
| Methanogenesis | 12.98% | 0.07% |
| **Methylamine/dimethylamine/trimethylamine => methane** | **19.54%** | **0.10%** |
| **Amino Acids and Derivatives** | **6.56%** | **0.03%** |
| Creatine and Creatinine Degradation | 6.56% | 0.03% |
| **Carbohydrates** | **12.98%** | **0.07%** |
| Methanogenesis | 12.98% | 0.07% |
| **Total** | **100.00%** | **0.52%** |

Table S3: Relative abundance of reads in the relevant methanogenesis pathways from WGS_whole dataset using the SEED database through MG‑RAST server.
