# Supplementary material for: Microbial diversity of a full‐scale UASB reactor applied to poultry slaughterhouse wastewater treatment: integration of 16S rRNA gene amplicon and shotgun metagenomic sequencing
Source: Microbiologyopen. 2017 Feb 23;6(3):e00443. doi: 10.1002/mbo3.443 (PMC5458456; doi:10.1002/mbo3.443)
Supplement: Supplementary file 4 [file MBO3-6-na-s004.docx]

| **Description** | **Nº of Reads** |
| --- | --- |
| **Bacitracin** | **1,388** |
| BACA | 1,388 |
| **Cephalosporin;Penicillin** | **2** |
| BL2B_TEM | 2 |
| **Cephalosporin;Penicillin;Penicillin;Cephalosporin_Ii;Cephalosporin_i** | **1** |
| BL2B_TEM1 | 1 |
| **Chloramphenicol;Fluoroquinolone** | **1** |
| BMR | 1 |
| **Cloxacillin;Penicillin** | **4** |
| BL2D_OXA9 | 4  Table S4: Distribution of reads among the 43 antibiotics-resistance genes (ARGs) types in the WGS_whole dataset against the ARDB database. |
| **Lincomycin** | **34** |
| LNUA | 6 |
| LNUB | 28 |
| **Lincosamide;Streptogramin_B;Macrolide** | **9** |
| ERMB | 5 |
| ERMC | 3 |
| ERMG | 1 |
| **Macrolide** | **29** |
| MEFA | 22 |
| MPHB | 7 |
| **Multidrug Resistance Efflux Pump.** | **4,057** |
| ACRB | 14 |
| ADEB | 3 |
| CEOB | 642 |
| MACB | 13 |
| MEXA | 2 |
| MEXB | 835 |
| MEXD | 2 |
| MEXE | 2 |
| MEXF | 2,520 |
| MEXX | 1 |
| OPCM | 2 |
| OPRM | 1 |
| OPRN | 5 |
| SMEB | 10 |
| SMEE | 5 |
| **Polymyxin** | **49** |
| ARNA | 49 |
| **Streptogramin_A** | **13** |
| VATB | 13 |
| **Streptomycin** | **2** |
| APH33IB | 1 |
| APH6ID | 1 |
| **Sulfonamide** | **10** |
| SUL1 | 10 |
| **Tobramycin;Kanamycin;Sisomicin;Dibekacin;Gentamicin** | **3** |
| ANT2IA | 3 |
| **Vancomycin** | **2** |
| VANRG | 2 |
| **Tetracycline** | **221** |
| TET32 | 11 |
| TETC | 3 |
| TETG | 1 |
| TETL | 15 |
| TETM | 28 |
| TETPA | 3 |
| TETPB | 3 |
| TETT | 155 |
| TETW | 2 |
